# A Novel Genus of Actinobacterial Tectiviridae

**DOI:** 10.3390/v11121134

**Published:** 2019-12-07

**Authors:** Steven M. Caruso, Tagide N. deCarvalho, Anthony Huynh, George Morcos, Nansen Kuo, Shabnam Parsa, Ivan Erill

**Affiliations:** 1 Department of Biological Sciences, University of Maryland Baltimore County (UMBC), Baltimore, MD 21250, USA; scaruso@umbc.edu (S.M.C.); ST33486@umbc.edu (A.H.); gmorcos1@umbc.edu (G.M.); nansen1@umbc.edu (N.K.); shab2@umbc.edu (S.P.); 2 Keith R. Porter Imaging Facility, University of Maryland Baltimore County (UMBC), Baltimore, MD 21250, USA; tagided@umbc.edu; 3 College of Natural and Mathematical Sciences, University of Maryland Baltimore County (UMBC), Baltimore, MD 21250, USA

**Keywords:** tectivirus, *Streptomyces*, actinobacteria, pathogen, plant, lipid membrane, capsid protein, potato scab

## Abstract

*Streptomyces phages WheeHeim* and *Forthebois* are two novel members of the *Tectiviridae* family. These phages were isolated on cultures of the plant pathogen *Streptomyces scabiei*, known for its worldwide economic impact on potato crops. Transmission electron microscopy showed viral particles with double-layered icosahedral capsids, and frequent instances of protruding nanotubes harboring a collar-like structure. Mass-spectrometry confirmed the presence of lipids in the virion, and serial purification of colonies from turbid plaques and immunity testing revealed that both phages are temperate. *Streptomyces* *phages WheeHeim* and *Forthebois* have linear dsDNA chromosomes (18,266 bp and 18,251 bp long, respectively) with the characteristic two-segment architecture of the *Tectiviridae*. Both genomes encode homologs of the canonical tectiviral proteins (major capsid protein, packaging ATPase and DNA polymerase), as well as PRD1-type virion-associated transglycosylase and membrane DNA delivery proteins. Comparative genomics and phylogenetic analyses firmly establish that these two phages, together with *Rhodococcus* *phage Toil*, form a new genus within the *Tectiviridae*, which we have tentatively named *Deltatectivirus*. The identification of a cohesive clade of Actinobacteria-infecting tectiviruses with conserved genome structure but with scant sequence similarity to members of other tectiviral genera confirms that the *Tectiviridae* are an ancient lineage infecting a broad range of bacterial hosts.

## 1. Introduction

The *Tectiviridae* are a family of tail-less double-stranded DNA (dsDNA) phages characterized by an internal protein-rich lipid membrane enclosed within a non-enveloped icosahedral proteinaceous capsid [1,2]. Tectiviruses bind to the host cell surface via receptor binding proteins integrated into the spikes that protrude from each capsid vertex [3]. After adsorbing to the host cell, the tectiviral membrane vesicle is reorganized to generate a tubular structure. This nanotube protrudes from one of the capsid vertices in order to inject the viral DNA [4,5]. DNA injection is presumed to be driven initially by pressure build-up during DNA packaging, and is contingent on the formation of the nanotube and the consequent reduction in membrane vesicle volume [5,6]. Tectiviruses are substantially underrepresented among known prokaryotic viruses, but together with other lipid membrane-containing viruses they have been shown to form an ancient and widely distributed lineage of viruses with important implications for prokaryotic biology and evolution [2,7]. The tectiviral lineage is characterized primarily by the double jelly-roll major capsid protein (DJR MCP) that makes up the viral capsid, and the use of a specialized FtsK-HerA superfamily ATPase for DNA packaging [8]. The *Tectiviridae* family is currently divided into three main genera [9]. The *Alphatectivirus* genus was defined based on the type species *Pseudomonas virus PRD1* and comprises a group of closely related virulent phages infecting a variety of Gammaproteobacteria hosts [1]. These include *Enterobacteria phage PR4* and *Enterobacteria phage PR3 Enterobacteria phage PR772* isolated, respectively, on *Escherichia coli* and *Proteus mirabilis* [10], as well as *Enterobacteria phage L17*. Extensive work on *Pseudomonas virus PRD1* has determined many of the morphological and genetic features used to define the family *Tectiviridae* [11].The *Betatectivirus* genus is exemplified by the *Bacillus virus Bam35* type species. It encompasses a broader group of temperate phages infecting members of several Firmicutes genera. These phages include *Bacillus phage pGIL01* and *Bacillus phage pGIL16*, infecting *Bacillus cereus* group hosts [12], as well as *Bacillus virus AP50* and *Bacillus phage Wip1*, capable of infecting the bacterial pathogen *Bacillus anthracis* [13,14]. Members of the *Betatectivirus* genus replicate independently of the host chromosome as linear plasmids and regulate transition to a lytic lifestyle via the host SOS transcription repressor LexA [15]. The *Gammatectivirus* genus contains only one known member, *Gluconobacter phage GC1*, which is also temperate and infects the Alphaproteobacterium *Gluconobacter cerinus* [16].

*Rhodococcus phage Toil*, a phage infecting *Rhodococcus opacus*, was recently characterized and reported as the first tectivirus capable of infecting Actinobacteria [17]. Here we report the isolation, characterization and genome sequencing of two new tectiviruses as part of an ongoing effort to characterize phages infecting the causative agent of potato scab, *Streptomyces scabiei*, within the Science Education Alliance-Phage Hunters Advancing Genomics and Evolutionary Science (SEA-PHAGES) and STEM BUILD at UMBC programs [18,19,20,21]. *S. scabiei* is best known for its worldwide economic impact on potato crops [22,23], but it has a broad host range that encompasses carrot and radish taproot crops, as well as many other dicots and monocots [22]. This plant pathogen produces the phytotoxin thaxtomin A, which inhibits cellulose synthesis, leading to the signature scab symptoms [24]. Because there is no known mechanism for effective disease suppression after initial infestation, multiple management strategies and approaches have been explored for the control of *S. scabiei* [22], including the effective use of virulent bacteriophages [25]. The novel tectiviruses reported in this work (*Streptomyces phage WheeHeim* and *Streptomyces phage Forthebois*) were isolated on *S. scabiei* and expand the existing collection of Caudovirales phages active against this important pathogen [21,25,26,27,28]. These two phages share morphological features, genomic structure and moderate sequence similarity with *Rhodococcus phage Toil*. Phylogenetic analyses clearly establish that *Rhodococcus phage Toil* and *Streptomyces phages WheeHeim* and *Forthebois* collectively define a monophyletic group of phages infecting Actinobacteria within the *Tectiviridae* family. We propose that, given their distinctive features, these three bacteriophages be considered the founding members of a novel *Tectiviridae* genus, tentatively named *Deltatectivirus*.

## 2. Materials and Methods

### 2.1. Isolation and Purification of Phages Infecting Streptomyces Scabiei

Bacterial strains and phages used in this study are listed in Table S1. *Streptomyces* cultures were grown in nutrient broth (BD Difco) supplemented with 10 mM MgCl_2_, 8 mM Ca(NO_3_)_2_, 0.5% glucose (NB^+^), and 0.05% polyethylene glycol (PEG 8000) at 30 °C for at least 48 h with shaking prior to use [29]. *Streptomyces phages WheeHeim* and *Forthebois* were extracted from soil samples with phage buffer (10 mM Tris pH 7.5, 10 mM MgSO_4_, 1 mM CaCl_2_, 68.5 mM NaCl) and filtered through a 0.22 μm filter. 500 μL of the filtrate was added to 250 µL of a 48 h culture of *Streptomyces scabiei* RL-34 (ATCC 49173), incubated 10 min at room temperature, combined with 4 mL of tryptic soy soft agar (BD), overlaid on nutrient agar (BD Difco) supplemented with 10 mM MgCl_2_, 8 mM Ca(NO_3_)_2_, and 0.5% glucose (NA^+^), and incubated for 24 to 48 h at 30 °C. Phages were plaque purified on lawns of *S. scabiei* as previously described [30].

### 2.2. Production of High-Titres Lysates and Phage Concentration

Crude stock and CsCl purified lysates were produced on NA^+^ solid media using protocols described previously [30]. After a minimum of three rounds of plaque purification, plaques were picked into phage buffer and diluted to produce plates with near-confluent lysis after infection of *S. scabiei*. Plates were then covered with 8 mL phage buffer, and incubated overnight at 4 °C. The lysate was centrifuged for 20 min at 2500× *g* and then passed through a 0.22 μm filter. For CsCl purification, 300 mL filtered crude lysate at 10^9^ pfu/mL was produced, concentrated by incubation with PEG 8000, and purified by two rounds of banding by CsCl ultracentrifugation [30].

### 2.3. Host Range Analysis

The host range of each phage was tested by spotting diluted crude lysate on lawns of *Streptomyces* spp. [31]. Cultures were obtained from the Agricultural Research Service (ARS, https://nrrl.ncaur.usda.gov/). NA^+^ plates were inoculated with 250 µL of 48 h cultures of *Streptomyces azureus* NRRL B-2655, *Streptomyces bobili* NRRL B-1338, *Streptomyces bottropensis* ISP-5262, *Streptomyces coelicolor* subsp. coelicolor NRRL B-2812, *Streptomyces coelicolor* subsp. coelicolor A3(2) NRRL B-16638, *Streptomyces diastatochromogenes* NRRL ISP-5449, *Streptomyces griseus* subsp. griseus NRRL B-2682, *Streptomyces mirabilis* NRRL B-2400, *Streptomyces neyagawaensis* ISP 5588, *Streptomyces xanthochromogenes* NRRL B-5410, and the control host *S. scabiei* in trypticase soy soft agar (Table S1). Once set, 5 µL aliquots of serially diluted lysate were spotted on each plate, then incubated at 30 °C for up to 96 h. Resulting plaques were re-purified on the test host to confirm phage amplification and exclude killing from without. Efficiency of plating (EOP) was calculated as the titer determined on the test species divided by the titer determined on the isolation host.

### 2.4. Isolation of Lysogens and Immunity Testing

Putative *WheeHeim* and *Forthebois* lysogens were isolated by spotting diluted crude lysate on NA^+^ overlaid with 250 µL of a 48 h culture of *S. scabiei* RL-34 in trypticase soy soft agar incubated for 96 h at 30 °C. Cells were isolated from a zone of clearing and streak purified three times. A resulting colony was then used to inoculate 3 mL NB^+^ with 0.05% polyethylene glycol (PEG 8000) and incubated for 48 h at 30 °C. To test for the presence of released phage, 500 µL of the culture was centrifuged for 1 min at 11,700× *g*, and the supernatant was serially diluted and 3 µL spotted on NA^+^ overlaid with 250 µL of a 48 h culture of *S. scabiei* RL-34 in trypticase soy soft agar incubated for 48 h at 30 °C. To test for superinfection immunity, crude lysate stocks of *Streptomyces phages Scap1* [21], *Forthebois*, *WheeHeim*, and *S. scabiei (Forthebois)* lysogen supernatant were serially diluted and 3 µL spotted on NA^+^ overlaid with 250 µL of a culture of *S. scabiei* RL-34 (*Forthebois*) lysogens in trypticase soy soft agar incubated for 96 h at 30 °C with daily examination.

### 2.5. Electron Microscopy

10 µL phage lysate was placed on a 200 mesh formvar-covered and carbon-coated copper grids (EMS) and allowed to set for 1 min, briefly rinsed with ultra-pure water, then stained with 2% uranyl acetate for 2 min. Phages were imaged on an Morgagni M268 Transmission Electron Microscope (FEI, Hillsboro, IL, USA) equipped with an Orius CCD camera (Gatan Inc., Pleasanton, CA, USA).

### 2.6. Extraction of Phage DNA

Five µL nuclease mix (0.25 mg/mL DNase I, 0.25 mg/mL RNase A, 50% glycerol, 150 mM NaCl) was added to 1 mL crude lysate, mixed gently by inversion, and incubated at 37 °C for 10 min. The nuclease was then inactivated and phage gDNA isolated by the addition of 15 µL 0.5 M EDTA, 50 µL 10% SDS, and 0.5 µL 20 mg/mL Proteinase K followed by washing and concentration using a Wizard DNA clean-up system (Promega, Madison, WI, USA). DNA was eluted in ddH_2_0 and quantified by Thermo Scientific nanodrop (Waltham, MA, USA) and gel electrophoresis.

### 2.7. DNA Sequencing

Sequencing was performed by the NC State Genomic Sciences Laboratory to approximately 10,000× coverage with the MiSeq platform (Illumina, San Diego, CA, USA). Assembly was performed using the CLC Genomics Workbench NGS de novo assembler (v6) with default settings and minimum contig length of 500 bp. Ends were determined from analysis of Illumina single-end sequencing reads by the Pittsburgh Bacteriophage Institute. Reads were examined for the presence of inverted repeats and for their presence of reads extending across contig ends.

### 2.8. Genome Annotation and Analysis

Genome annotation was completed using DNA Master (v5.23.3) [32] with default settings. Automated gene calls were independently assessed by two different annotators and further validated with complemented ARAGORN (v1.2.38), tRNAscan (v2.0) and GeneMarkS (v3.25) [33,34,35]. Functional annotations were performed using the NCBI BLASTP and HHPred services with default parameters following the SEA-PHAGES annotation guidelines (maximum BLASTP *e*-value: 10^−7^, minimum HHpred probability: 90%) and independently assessed by at least two annotators [36,37,38]. A collection of experimentally reported LexA-binding sites in Actinobacteria was downloaded from the CollecTF database [39]. These sites were used to scan the *Streptomyces phages WheeHeim* and *Forthebois* genome sequences using XFITOM [40]. Pair-wise analyses of genome-wide nucleotide and amino acid similarity were computed using Average Amino acid Identity (AAI) and Average Nucleotide Identity (ANI) calculators [41].

### 2.9. Mass Spectroscopy

CsCl purified samples of *Streptomyces phage WheeHeim* and *Mycobacterium phage Rosebush* [42] were dialyzed into water-methanol (50:50 *v*/*v*) with 0.1% Formic acid using a 3.5 K MWCO Pierce Slide-A-Lyzer MINI Dialysis Device (Thermo Scientific, Waltham, MA, USA). Viral particles were analyzed by an Autoflex MALDI-TOF/MS (Bruker, Billerica, MA, USA) in negative mode [43] using 2,5-Dihydroxybenzoic acid (DHB) as the matrix.

### 2.10. Ortholog Detection

Orthologous protein sequences among *Rhodococcus phage Toil* and *Streptomyces phages WheeHeim* and *Forthebois* were determined as best-reciprocal BLAST hits with an *e*-value cutoff of 1 × 10^−5^. To detect more distant orthologs, Hidden Markov Models (HMM) were downloaded for the PFAM (31.0), COG (2014) and eggNOG (bactNOG and Viruses; 4.5.1) databases [44,45,46,47] and searched with phage proteins using *hmmscan* [48] with an *e*-value cutoff of 1 × 10^−5^. Pre-compiled multiple sequence alignments for signature protein families present in DJR MCP-containing viruses were obtained from [8], processed into HMM with hmmbuild and used to search phage protein files with *hmmsearch* and a 1 × 10^−10^ *e*-value cutoff.

### 2.11. Phylogenetic Methods

Nucleotide and protein sequences for *Tectiviridae* phages were obtained from the NCBI GenBank and RefSeq databases [49]. Protein sequences were aligned using M-COFFEE with default parameters [50], and the resulting alignments were pruned with Gblocks with less astringent selection options [51]. Pruned alignments were used for Bayesian phylogenetic inference with MrBayes 3.1 applying a mixed four-category Gamma distributed rate model plus proportion of invariable sites model (invgamma) [52]. Two Metropolis-Coupled Markov Chain Monte Carlo runs with four independent chains were carried out for 5 × 10^5^ generations. The resulting consensus trees were plotted with Dendroscope [53]. A genome-based phylogeny was generated with the VICTOR webservice [54]. Intergenomic protein sequence distances were computed with 100 pseudo-bootstrap replicates using the Genome-BLAST Distance Phylogeny (GBDP) method optimized (distance formula d_6_) for prokaryotic viruses [54,55], and a minimum evolution tree was computed with FASTME on the resulting intergenomic distances [56].

## 3. Results

### 3.1. Isolation, Host Range and Morphological Characterization of Streptomyces Phages WheeHeim and Forthebois

*Streptomyces phages WheeHeim* and *Forthebois* were isolated on a pure culture of *S. scabiei* RL-34 from soil samples collected, respectively, in Saigon (Vietnam) and Halethorpe (MD, USA) by undergraduate students participating in the SEA-PHAGES program. *S. scabiei* RL-34 is the type strain for the causative agent of potato scab, and forms heavily branched sporulating aerial mycelia in agar media [57]. The SEA-PHAGES program has to date isolated 56 additional phages capable of infecting *S. scabiei* RL-34. These phages all display caudoviral morphologies and a lytic lifestyle [58]. *Streptomyces phages WheeHeim* and *Forthebois*, in contrast, generated turbid, circular plaques with 2–5 mm diameter on lawns of *S. scabiei* RL-34 after 48 h at 30 °C. The host range of both phages was assessed by spotting diluted crude lysate on lawns of ten additional *Streptomyces* species. Both phages displayed a fairly limited host range among the tested strains, being able to infect only *S. mirabilis* NRRL B-2400 at an efficiency-of-plating (EOP) of approximately 20, in addition to the isolation host *S. scabiei* RL-34 (Table S2). Phages infecting only *S. mirabilis* and *S. scabiei* among this group of tested strains have been reported previously for *Siphoviridae* phages infecting *S. scabiei* [21], suggesting that *S. mirabilis* and *S. scabiei* share important properties for phage infection that are absent in the closely related *S. diastatochromogenes* [59].

**Figure 1:**
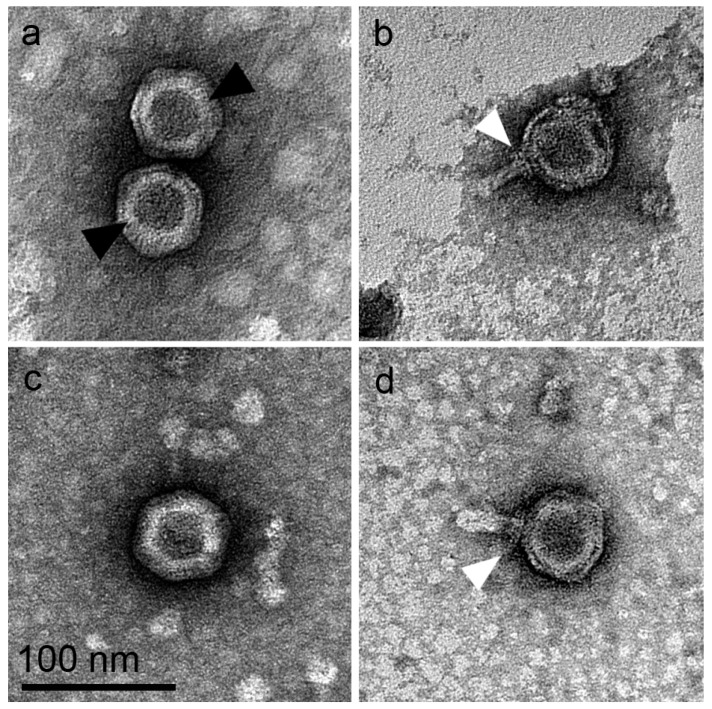
Representative TEM images of (**a**) CsCl-purified *WheeHeim*; (**b**) *WheeHeim* with nanotube from crude lysate; (**c**) *Forthebois* from crude lysate; (**d**) *Forthebois* with nanotube from crude lysate. Black arrowheads indicate lipid membrane. White arrowheads indicate collar-like structure. Scale bar = 100 nm for all panels.

Transmission electron microscopy (TEM) images of *WheeHeim* and *Forthebois* revealed viral particles with six-sided, double-layered phage capsids (Figure 1a,c; black arrows) with an average circumscribed diameter of 65.5 nm (SD = 4.4, *n* = 20) and 64.4 nm (SD = 4.5, *n* = 20), respectively, consistent with the two-dimensional projection of an icosahedral structure. Several virion particles demonstrated protruding nanotubes with a respective average length of 37.0 nm (*WheeHeim*; SD = 4.4, *n* = 13) and 41.6 nm (*Forthebois*; SD = 8.4, *n* = 170). Examination of hundreds of virions revealed that these tubular structures were present on 3–7% of virions in the crude lysate (*WheeHeim n* = 6/200; *Forthebois n* = 14/186), protruding in all cases from an apparent capsid vertex, consistent with previous reports of tectiviral particles [6,60,61,62] (Figure 1b,d; white arrows). Together, the presence of a double-layered membrane and of protruding nanotubes strongly suggested that these bacteriophages belonged to the *Tectiviridae* family. In virions with protruding tubular structures, a reduction of ~4.0% in capsid size was observed, consistent with previous reports [6], and a collar-like structure surrounding the base of the tube could also be discerned (Figure 1b,d and Figure S1). Tail collar structures have been described in members of all families of Caudovirales [63,64,65], but had to date not been reported in the *Tectiviridae*. A re-examination of previously reported tectiviral nanotube images suggests that these structures may be present in other *Tectiviridae* [61,62], and may therefore be a common feature of tectiviral particles with protruding nanotubes.

### 3.2. Genome Sequencing and Annotation

To gain further insight into *Streptomyces phages WheeHeim* and *Forthebois*, DNA from both phages was extracted and sequenced on an Illumina MiSeq Next Generation Sequencer. The chromosomes of both phages were determined by read analysis to consist of a linear dsDNA molecule of 18,266 bp (*WheeHeim*) and 18,251 bp (*Forthebois*). Both phages presented 24 bp inverted repeats at their ends, with no reads extending past the contig ends. This arrangement is reminiscent of φ29-like phages, which are typically packaged with covalently-linked terminal proteins [66]. In spite of their distant collection sites, the *WheeHeim* and *Forthebois* chromosomes present an average nucleotide identity (ANI) of 88.5% [67] and a very similar GC content (54.6% and 53.6%, respectively). The *WheeHeim* and *Forthebois* chromosomes contain 36 protein coding genes, as well as a tRNA-Asn gene. Function could be assigned with confidence to only half of the genes (Table 1). Both genomes present the same overall two-segment arrangement, with a first segment of genes in the reverse strand and a second, larger one, in the forward strand. This arrangement is reminiscent of the one reported recently for *Rhodococcus phage Toil* [17]. The first segment encompasses genes coding for a DNA polymerase, a single-stranded DNA binding protein and a homolog of the nucleotide pyrophosphohydrolase MazG [68]. The second, larger segment in the forward strand contains structural genes and genes involved in cell lysis, such as, respectively, a homolog of the PRD1 major capsid protein and a LysM-like Endolysin [69]. This segment also encompasses two homologs of the PRD1 P34 membrane DNA delivery protein, a hydrolase and a glycosyltransferase. The identification of two homologs of *Pseudomonas virus PRD1* proteins, and the similarity of the two-segment genome organization to that reported for *Rhodococcus phage Toil*, supported the morphological inference that *Streptomyces phages WheeHeim* and *Forthebois* are tectiviruses.

### 3.3. Virion Stability, Lifestyle and Determination of the Membrane Composition

**Figure 2:**
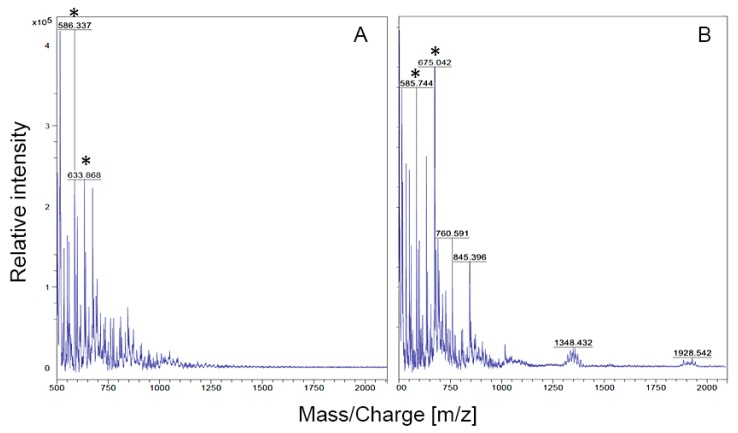
MALDI-TOF spectra for (**A**) *Mycobacterium phage Rosebush* and (**B**) *Streptomyces phage WheeHeim*. Asterisks denote DHB (2,5-Dihydroxybenzoic acid) matrix peaks. m/z denotes mass (m) to charge (z) ratio.

The presence of an internal protein-rich lipid membrane is a hallmark of tectiviruses. To ascertain whether the double-layered phage capsids observed in TEM images corresponded to an internal lipid membrane enclosed within a non-enveloped capsid, CsCl purified samples of *Streptomyces* phage *WheeHeim* were analyzed by MALDI-TOF, using *Mycobacterium phage Rosebush*, a siphovirus, as a control (Figure 2) [42]. The *WheeHeim* spectrum is enriched for peaks likely corresponding to phosphatidylglycerol (PG; *m*/*z* 845) and phosphatidylethanolamine (PE; *m*/*z* 760), known to be major components of the lipid membrane in tectiviruses infecting both Gram-positive and Gram-negative hosts [43,70,71]. Furthermore, the *WheeHeim* spectrum also presents multiple peaks in the *m*/*z* 1300–1500 range, corresponding to PG dimers (cardiolipins; CL). These peaks, and specifically the center peak at *m*/*z* 1348, match those reported for *Pseudomonas virus PRD1* [43]. Overall, the *WheeHeim* spectrum, modulated by its host lipid species, is consistent with the lipid composition of previously reported tectiviruses [71,72].

**Figure 3:**
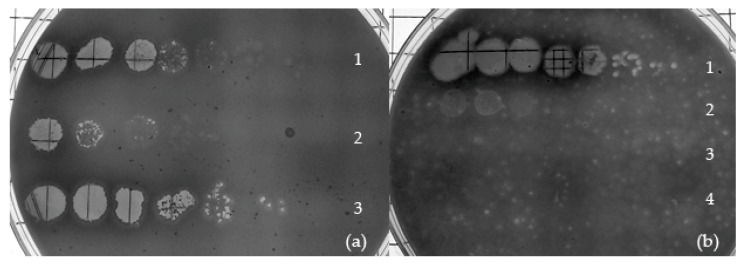
Lysogen isolation and testing. (**a**) Liquid phage release from *WheeHeim* lysogens. Serially diluted stock *Streptomyces phage WheeHeim* (**row 1**), streak purified *S. scabiei* (*WheeHeim*) lysogen supernatant (**row 2**), and stock *Streptomyces phage Forthebois* (**row 3**) spotted on NA^+^ overlayed with *S. scabiei* RL-34 after 48 h at 30 °C. (**b**) Superinfection immunity test of *S. scabiei* (*Forthebois*) lysogen. Serially diluted crude lysate stocks of *Streptomyces phage Scap1* (**row 1**) *Forthebois* (**row 2**), *WheeHeim* (**row 3**), and *S. scabiei* (*Forthebois*) lysogen supernatant (**row 4**) spotted on NA^+^ overlayed with *S. scabiei* (*Forthebois*) lysogen after 48 h at 30 °C.

*Streptomyces phages WheeHeim* and *Forthebois* presented turbid plaques, which can be indicative of lysogeny. Most tectiviruses infecting Gram-positive hosts have been shown to be temperate [15,73], thus we attempted to isolate lysogens by isolating and serially purifying cells that survived phage infection, then testing for the presence of spontaneous release of phage. Our results show that, after purification, *S. scabiei* cells isolated from apparent colonies within turbid plaques were able to induce plaque formation on a *S. scabiei* RL-34 lawn, indicating that they are releasing phage (Figure 3a and Figure S2).

Furthermore, lawns of these putative *S. scabiei* lysogens could be infected by *Streptomyces phage Scap1* (a *Siphoviridae* infecting *S. scabiei* [21]), but not by *WheeHeim* or *Forthebois*, indicating that lysogens exhibited superinfection exclusion (Figure 3b). *Bacillus*-infecting tectiviruses have been reported to use the host’s LexA repressor to activate the lytic cycle by binding at multiple promoters [15,74], but no putative LexA-binding sites could be detected on genome-wide scans of *Streptomyces phages WheeHeim* and *Forthebois* genome sequences. Similarly, none of the lysogeny associated proteins from *Bacillus*-infecting tectiviruses could be detected in *Streptomyces phages WheeHeim* and *Forthebois*. These results suggest that *Streptomyces phages WheeHeim* and *Forthebois* are temperate phages. Alternatively, they might be plasmid-phages with an unusually long carrier state capable of exhibiting superinfection exclusion after more than 100 generations [75,76]. In both cases, the absence of homologs for conventional lysogeny-related genes or plasmid-associated *rep* genes puts forward the hypothesis that these phages might utilize a novel mechanism for determining phage replication and cell fate.

### 3.4. Comparative Genomics and Phylogenetic Analysis

Genomic analyses of previously reported *Tectiviridae* phages have established the family-wide conservation of three major proteins: a double jelly-roll major capsid protein (DJR MCP), a FtsK-HerA superfamily DNA packaging ATPase and a protein-primed family B DNA polymerase [1,8,15]. These studies have also revealed that genome organization among tectiviruses is highly conserved, in spite of low sequence conservation. To thoroughly investigate the relationship of *Streptomyces phages WheeHeim* and *Forthebois* to previously described *Tectiviridae* phages we first analyzed best-reciprocal BLAST hits with *Rhodococcus phage Toil*, the only phage for which a BLASTP against the NCBI NR database would return significant results. This analysis revealed significant similarity between six genes across these three phages (Table 2). These include the aforementioned major capsid protein, packaging ATPase and DNA polymerase, as well as the PRD1-like membrane DNA delivery protein (duplicated in *Streptomyces phages WheeHeim* and *Forthebois*), the entry lysozyme (Toil_gp23) and a hypothetical protein (Toil_gp11). To detect more distant homologs, we conducted HMM-based searches against these three phages and representative phages from previously described tectiviral genera (*Pseudomonas virus PRD1*, *Bacillus virus Bam35* and *Gluconobacter phage GC1*). These searches confirmed that the three core *Tectiviridae* proteins (MCP, ATPase and DNA polymerase) are conserved in these three phages and in all previously described *Tectiviridae* genera. They also identified ample conservation of a virion-associated transglycosylase (SEA_WHEEHEIM_26) known to be involved in PRD1 phage infection [77], and the presence in *Streptomyces phages WheeHeim* and *Forthebois* of a homolog of the M23 family metallopeptidase associated with the group of rolling circularly-replicating ssDNA viruses represented by *Flavobacterium* phage FLiP [8].

**Figure 4:**
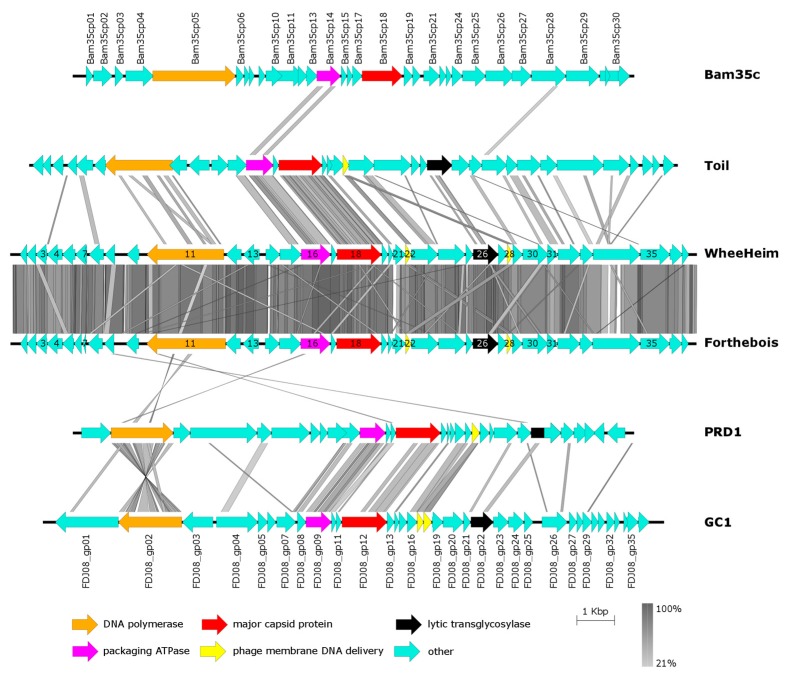
Genome maps of *Rhodococcus phage Toil*, *Streptomyces phages WheeHeim* and *Forthebois*, *Gluconobacter phage GC1*, *Pseudomonas virus PRD1* and *Bacillus virus Bam35*. Colored arrows indicate genes. Vertical bars indicate percent amino acid identity from pairwise tBLASTX comparisons on a grey scale. For genes with assigned function in Table 1, gene numbers for *Streptomyces phages WheeHeim* and *Forthebois* are superimposed on the corresponding colored arrows.

Having established distant orthology with proteins in other *Tectiviridae* genomes, we assessed the apparent similarity in genome organization between *Rhodococcus phage Toil* and *Streptomyces phages WheeHeim* and *Forthebois* in the broader context of *Tectiviridae* genome architecture. The genome maps displayed in Figure 4 reveal the previously noted structural genome similarity among the three established tectiviral genera [16], and show that this structural similarity extends to the *Rhodococcus* and *Streptomyces* phages. All genomes contain a first genomic segment that appears to have switched orientation in multiple instances, with the family B DNA polymerase as its only identifiable conserved element. The second segment, consistently oriented in the forward strand, encompasses the signature double jelly-roll major capsid protein and the DNA packaging ATPase, as well as phage membrane DNA delivery proteins and a lytic transglycosylase. The plot also illustrates the lack of substantial sequence conservation between members the three different genera of the family *Tectiviridae*. In contrast, and as observed for different tectiviral genera [1,15], *Rhodococcus phage Toil* and *Streptomyces phages WheeHeim* and *Forthebois* display a high degree of sequence similarity (Figure 4). The Average Amino Acid Identity (AAI) between *Rhodococcus phage Toil* and *WheeHeim* and *Forthebois* is, respectively, 35.3% and 39.1%, suggesting that these three phages are representatives of a single genus. In addition, the large ANI between *Streptomyces phages WheeHeim* and *Forthebois* (88.5%) indicates that they constitute separate species according to the species demarcation criteria of the International Committee on Taxonomy of Viruses (ICTV) [78,79].

**Figure 5:**
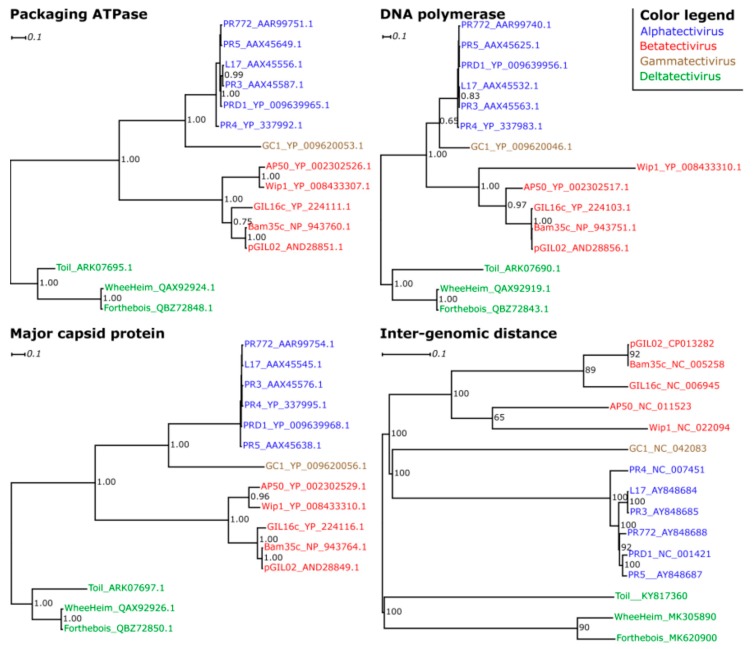
Phylogenetic trees resulting from Bayesian inference on multiple sequence alignments of the packaging ATPase, the DNA polymerase and the major capsid protein sequences, and from minimal evolution inference on BLAST-derived inter-genomic distances. Only support values above 0.5 posterior probability or 50% bootstrap support are shown. Trees were rooted arbitrarily for display purposes. Colors denote the previously described *Tectiviridae* genera (Alphatectivirus, blue; Betatectivirus, red; Gammatectivirus, brown) as well as the proposed novel genus Deltatectivirus (green).

To more rigorously ascertain whether *Rhodococcus phage Toil* and *Streptomyces phages WheeHeim* and *Forthebois* define a novel genus in the family *Tectiviridae*, we performed Bayesian phylogenetic inference with the aligned sequences of the three conserved *Tectiviridae* proteins (MCP, ATPase and DNA polymerase), as well as bootstrapped minimal evolution phylogenetic inference with intergenomic protein sequence distances inferred from pair-wise genome-wide tBLASTX. The independently inferred phylogenies (Figure 5) consistently and robustly place *Rhodococcus phage Toil* and *Streptomyces phages WheeHeim* and *Forthebois* in a cohesive cluster similarly distant from the previously reported genera within the family *Tectiviridae* as these are from each other, strongly supporting their classification as a new genus within the *Tectiviridae*. Following the established convention for the family, we have tentatively named this novel genus *Deltatectivirus*. The inferred phylogenies also conclusively cluster the *Alphatectivirus* and *Gammatectivirus* genera together, suggesting that these two groups arose from a common ancestor. The phylogenies indicate that the common ancestor of extant *Tectiviridae* species emerged at least one billion years ago, prior to the split between Gram-positive and Gram-negative bacteria [80,81]. The consistency between single-gene and whole genome phylogenies (Figure 5) and the conserved genome structure (Figure 4) suggests that tectiviruses have since diversified along with their hosts, rather than evolving through lateral gene transfer events enabling the swap of spike complexes to target novel hosts.

## 4. Conclusions

This work describes the morphological and genomic characterization of two novel phages infecting *Streptomyces scabiei*. Based on both morphological and genomic characteristics, we have established that *Streptomyces phages WheeHeim* and *Forthebois* should belong to the *Tectiviridae* family, wherein they could conform a distinct genus, tentatively named *Deltatectivirus*, that would include also the recently reported *Rhodococcus phage Toil*. Like other Gram-positive infecting tectiviruses, these phages display a lysogenic cycle, but the mechanism by which they mediate transition to the lytic cycle remains to be elucidated. This work also identifies for the first time the presence of a collar-like structure in the protruding nanotubes of tectiviruses, and provides strong support to the notion that the *Tectiviridae* define a family of bacteriophages with cohesive genome structure that originated well before the split of their Gram-positive and Gram-negative hosts.

## Figures and Tables

**Table 1:** Genes with functionally annotated products in *Streptomyces phages WheeHeim* and *Forthebois*.

Locus Tag	Locus Tag	Product ^1^
SEA_WHEEHEIM_3	SEA_FORTHEBOIS_3	MazG-like nucleotide pyrophosphohydrolase
SEA_WHEEHEIM_4	SEA_FORTHEBOIS_4	membrane protein
SEA_WHEEHEIM_7	SEA_FORTHEBOIS_7	tRNA-Asn
SEA_WHEEHEIM_11	SEA_FORTHEBOIS_11	DNA polymerase
SEA_WHEEHEIM_13	SEA_FORTHEBOIS_13	ssDNA binding protein
SEA_WHEEHEIM_16	SEA_FORTHEBOIS_16	hydrolase
SEA_WHEEHEIM_18	SEA_FORTHEBOIS_18	major capsid protein
SEA_WHEEHEIM_19	SEA_FORTHEBOIS_19	membrane protein
SEA_WHEEHEIM_20	SEA_FORTHEBOIS_20	membrane protein
SEA_WHEEHEIM_21	SEA_FORTHEBOIS_21	membrane protein
SEA_WHEEHEIM_22	SEA_FORTHEBOIS_22	membrane DNA delivery protein
SEA_WHEEHEIM_26	SEA_FORTHEBOIS_26	glycosyltransferase
SEA_WHEEHEIM_27	SEA_FORTHEBOIS_27	membrane protein
SEA_WHEEHEIM_28	SEA_FORTHEBOIS_28	membrane DNA delivery protein
SEA_WHEEHEIM_31	SEA_FORTHEBOIS_30	peptidase
SEA_WHEEHEIM_32	SEA_FORTHEBOIS_31	membrane protein
SEA_WHEEHEIM_36	SEA_FORTHEBOIS_35	LysM-like endolysin

^1^ Product functions annotated according to SEA-PHAGES functional annotation standards.

**Table 2:** Orthologs of *Streptomyces phages WheeHeim* and *Forthebois* proteins.

Inferred Function	Model	SEA_WHEEHEIM	SEA_FORTHEBOIS	Toil	Bam35	GC1 [FDJ08]	PRD1
DNA polymerase	ENOG4108SKE	11	11	gp07	cp05	gp02	02
	PF03175.13						
	DNAp						
	ENOG411ENY2						
	COG0417						
	ENOG4105CQ2						
hydrolase	ENOG4108JJ8	16	16	gp12	cp14	gp09	09
	COG3451						
	COG0433						
	ENOG411EP64						
	STIV_ATP						
	ENOG4105SBY						
	ENOG4107MA1						
major capsid protein	Bam_MCP	18	18	gp14	cp18	gp12	12
	MCP						
	PF09018.11						
membrane DNA delivery	PF11087.8	22	22	gp18	-	gp17	16
	PF11087.8	28	28	gp18	-	gp18	16
glycosyltransferase	ENOG4107N29	26	26	gp23	-	gp22	20
	ENOG41065RS						
	COG0741						
	PF01464.20						
	STIV_lysozyme						
	ENOG41090WR						
	ENOG4105E4C						
	ENOG411EP35						
hypothetical protein	PF01476.20	24	24	gp20	-	-	-
	ENOG4108I7Y						
	COG1652						
hypothetical protein	-	15	15	gp11	-	-	-
ssDNA binding protein	ENOG4107YH6	13	13	-	-	-	-
	COG0629						
	PF00436.25						
peptidase	ENOG4105DR5	31	30	-	-	-	-
	COG0739						
	PF01551.22						
	peptidase_M23						
MazG-like nucleotide pyrophosphohydrolase	ENOG4106A27	3	3	-	-	-	-
	COG1694						
LysM-like endolysin	ENOG4105RG6	36	35	-	-	-	-
	COG3023						
	PF01510.25						
membrane protein	ENOG4106BPY	4	4	-	-	-	-
NlpC family hydrolase	ENOG4105K4H	-	-	gp26	-	-	-
	COG0791						
	PF00877.19						
endolysin	ENOG4105SQZ	-	-	gp31	-	-	-
	PF13539.6						

Orthologs among *Rhodococcus phage Toil* and *Streptomyces phages WheeHeim* and *Forthebois* were determined as best-reciprocal BLAST hits. Only genes with significant (*e*-value < 1 × 10^−5^) *hmmscan/hmmsearch* matches to PFAM, EGGNOG, COG and previously published models are reported. Proteins matching the same model were considered to belong to the same orthologous group. An extended version of this table containing the search e-values is available in Table S3.

## Supplementary Materials

The following are available online at https://www.mdpi.com/1999-4915/11/12/1134/s1.
