# Supplementary material for: A Novel Genus of Actinobacterial Tectiviridae
Source: Viruses. 2019 Dec 7;11(12):1134. doi: 10.3390/v11121134 (PMC6950372; doi:10.3390/v11121134)
Supplement: Supplementary file 1 [file viruses-11-01134-s001.zip › Table_S1.pdf]

Table S1. Strains used in this study

**Bacterial strains**	**Source**
Streptomyces scabiei RL-34 ATCC 49173	ATCC
Streptomyces bobili NRRL B-1338	ARS
Streptomyces bottropensis ISP-5262	ARS
Streptomyces coelicolor subsp. coelicolor NRRL B-2812	ARS
Streptomyces coelicolor subsp. coelicolor A3(2) NRRL B-16638	ARS
Streptomyces diastatochromogenes NRRL ISP-5449	ARS
Streptomyces griseus subsp. griseus NRRL B-2682	ARS
Streptomyces mirabilis NRRL B-2400	ARS
Streptomyces neyagawaensis ISP-5588	ARS
Streptomyces xanthochromogenes NRRL B-5410	ARS
**Bacteriophage**	**GenBank Accession, PMID**
Mycobacterium phage Rosebush	AY129334, PMID:12705866
Streptomyces phage Forthebois	MK620900
Streptomyces phage Scap1	MF975637, PMID: 30533665
Streptomyces phage WheeHeim	MK305890
