# Supplementary material for: A Novel Genus of Actinobacterial Tectiviridae
Source: Viruses. 2019 Dec 7;11(12):1134. doi: 10.3390/v11121134 (PMC6950372; doi:10.3390/v11121134)
Supplement: Supplementary file 1 [file viruses-11-01134-s001.zip › Table_S2.pdf]

**Table S2. Host range of *Forthebois* and *WheeHeim***

| | Efficiency of Plating (EOP) | |
|---|---|---|
| **Bacterial strain** | ***Forthebois**** | ***WheeHeim**** |
| *Streptomyces bobili* NRRL B-1338 | - | - |
| *Streptomyces bottropensis* ISP-5262 | - | - |
| *Streptomyces coelicolor subsp. coelicolor* NRRL B-2812 | - | - |
| *Streptomyces coelicolor subsp. coelicolor* A3(2) NRRL B-16638 | - | - |
| *Streptomyces diastatochromogenes* NRRL ISP-5449 | - | - |
| *Streptomyces griseus subsp. griseus* NRRL B-2682 | - | - |
| *Streptomyces mirabilis* NRRL B-2400 | 30.3 | 20 |
| *Streptomyces neyagawaensis* ISP-5588 | - | - |
| *Streptomyces xanthochromogenes* NRRL B-5410 | - | - |

* The - symbol indicates not susceptible to phage
