# Supplementary material for: A Novel Genus of Actinobacterial Tectiviridae
Source: Viruses. 2019 Dec 7;11(12):1134. doi: 10.3390/v11121134 (PMC6950372; doi:10.3390/v11121134)
Supplement: Supplementary file 1 [file viruses-11-01134-s001.zip › Figure_S1.pdf]

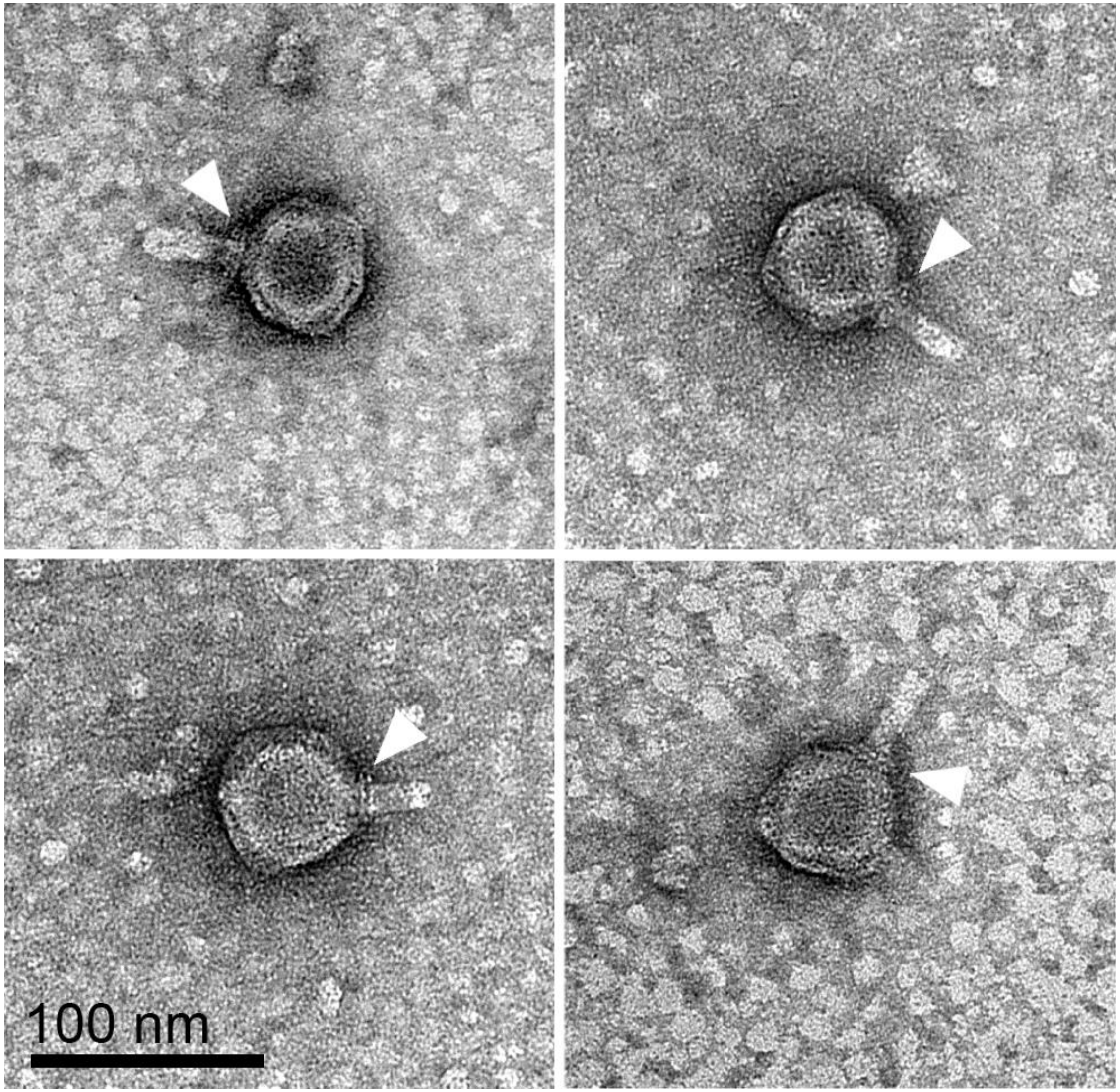

Figure S1. Representative TEM images of collars on protruding nanotubes from *Streptomyces* phage *Forthebois*. White arrowheads indicate collar-like structure. Scale bar = 100 nm for all panels.
