# Supplementary material for: A Novel Genus of Actinobacterial Tectiviridae
Source: Viruses. 2019 Dec 7;11(12):1134. doi: 10.3390/v11121134 (PMC6950372; doi:10.3390/v11121134)
Supplement: Supplementary file 1 [file viruses-11-01134-s001.zip › Figure_S2.pdf]

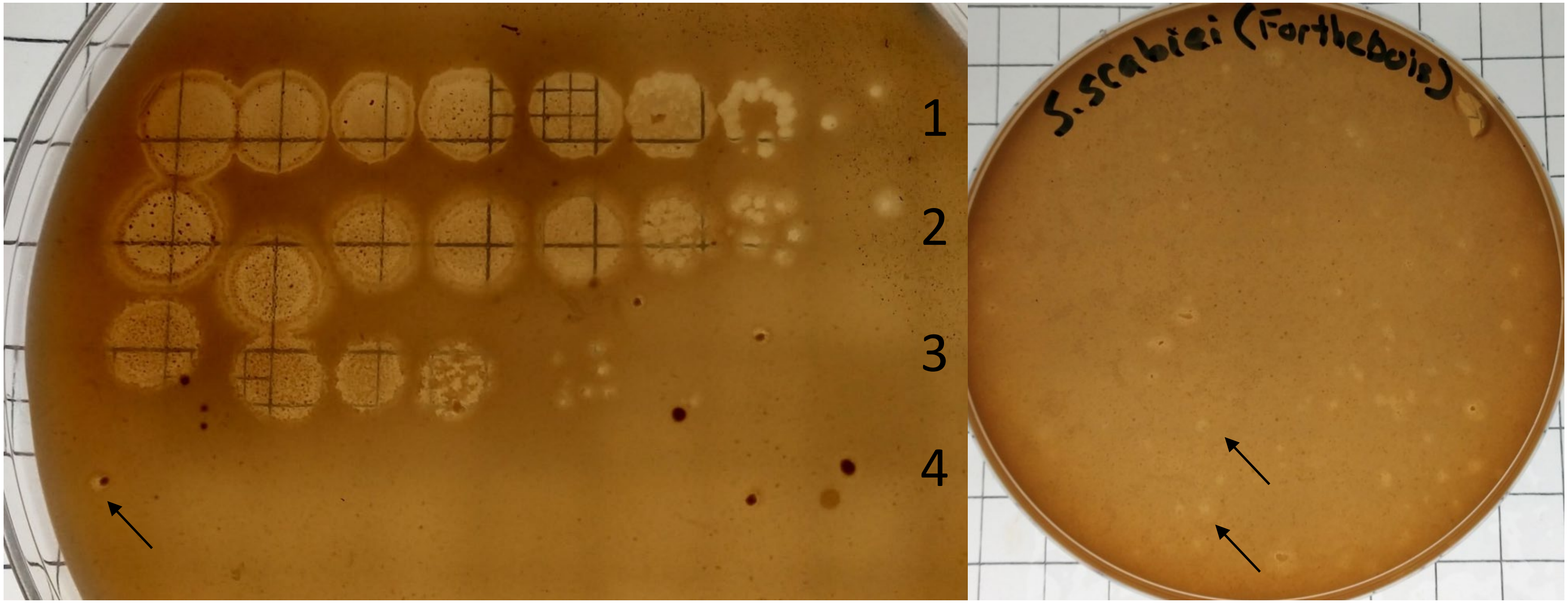

Figure S2. Stable *S. scabiei* (*Forthebois*) lysogen isolation and testing for production. **(a)** Liquid phage release from *Forthebois* lysogen. Serially diluted crude lysate stocks of *Streptomyces* phage *Scap1* (**row 1**), *Forthebois* (**row 2**), *WheeHeim* (**row 3**), and purified *S. scabiei* (*Forthebois*) lysogen supernatant (**row 4**) spotted on NA+ overlayed with *S. scabiei* after 48 hrs at 30 °C. Arrow indicates *Forthebois* plaque. **(b)** Lawn of *S. scabiei* (*Forthebois*) lysogen overlayed on NA+ after 48 hrs at 30 °C. Arrows indicate examples of spontaneous *Forthebois* plaques.
